# Latent factors on the design and adoption of gamified apps in primary education

**DOI:** 10.1007/s10639-023-11797-3

**Published:** 2023-04-25

**Authors:** Esteban Vázquez-Cano, María-Pilar Quicios-García, Javier Fombona, Jorge Rodríguez-Arce

**Affiliations:** 1 Faculty of Education, Universidad Nacional de Educación a Distancia, UNED, Room 2.15. C/ Juan del Rosal, 14, 28040 Madrid, Spain; 2 Universidad de Oviedo, Oviedo, Spain; 3 Universidad Autónoma del Estado de México, Toluca, México

**Keywords:** App, Gamification, Curriculum, Satisfaction, Efficacy, Primary education

## Abstract

The main objective of this research is to determine the perception of teachers about the elements that increases the educational effectiveness of gamified apps in primary education. A methodology based on an importance-performance analysis was daeveloped, using a structural equations model to calcuate the degree of importance of each variable. The sample was formed of 212 Spanish teachers with experience using educational apps in the teaching–learning process. Six categories were identified as precursors of educational effectiveness: (1) curriculum connection, (2) feedback and operational experience, (3) assessment and learning analytics, (4) sustainability (Protection Personal data), (5) equal access and (6) flow. These six categories enhance the three traditional areas of gamification intervention: cognitive, emotional and social. In this sense, the design and adoption of an educational gamified app should: (1) establish a clear link between the game and curricular content and competence development; (2) promote self-regulated learning through individual and collaborative activities; (3) offer adapted learning by integrating differentiated personalized learning pathways; (4) integrate learning analytics that can be consulted by teacher, student and family; (5) comply with data protection regulation and promote a safe, sustainable and ethical use of the information generated; (6) take into account different levels of functional diversity. When the gamified app design incorporates these attributes, primary education teachers perceive that such resources can be integrated effectively into the teaching–learning processes.

## Introduction

Gamified educational apps are being used more and more in the different stages of education. These apps can now be adapted to content established by the teacher, for example, apps to create quizzes and questionnaires, such as Kahoot, or apps desigend for micro-content in, for example, musical composition (Bloom), geometry (Geogebra), anatomy (Anatomy Learning) and English at various levels (“Duolingo” or “LearnEnglish Kids: Playtime”) and still more aimed at developing general content, with different grades of difficulty and gamified layouts (“Academons” or “Montessori Monster Math Lab”). The proposal of these educational apps ranges far and wide, and their turnover is rapid. The problem with such apps is that they are generally developed by medium-to large-scale companies whose prime objective is commercial, and whose designs rarely take into account the curricular content and competences for students across all levels of pre-university education, especially in primary education. Many teachers are reluctant to use educational apps for specific tasks in the classroom for reasons such as a lack of training in their use (Da Silva et al., 2019), a percieved lack of curriculum connection (Hirsh-Pasek et al., 2015a, b) and of digital sustainability (security, ethical use and protection of data of minors) (Vázquez-Cano et al., 2022). At the same time, parents are increasingly turning to these apps to encourage their children to use their mobile devices for educational purposes; such apps combine education and entertainment, though often lacking clear criteria on curricular content, the recommended time to be spent on the app, the type of game it is (individual or social) or the premises relating to their download and usage (Jong, 2019). It is important to highlight that the Spanish education system in its recent law of education (enacted in 2020) has made a firm commitment to the integration of technology in primary education with the proposal of learning situations in which the digital component and the inclusion of educational apps constitute a fundamental resource for the development of key competencies in and out of school. In this sense, within the Spanish educational scenario, the use of digital devices and educational apps in the classroom is allowed as long as it has a clear curricular content and serves for the development of the objectives of primary education.

In line with these considerations, the main objective of this research is to determine the perception of teachers about the elements that increases the educational effectiveness of free gamified apps in primary education. The analysis of the determining variables for the effectiveness of an educational app from the perspective of teachers can provide key elements for designers, as well as for the suitable selection of educational apps by teachers. In this sense, a close collaboration between teachers and app designers can help to generate new and more sustainable formats, linked to the curriculum and assessment procedures.

## Variables in the design and adoption of gamified apps

Digital game-based learning (DGBL) is a growing trend in education today, and there is increasing scientific evidence that DGBL both inside and outside the classroom can raise students’ academic performance (Clark et al., 2016). For this to happen, the device and type of game must comply with a set of techno-didactic parameters that promote learning and make the gaming experience both motivating and challenging. Among the various resources available for the teaching–learning processes, gamified apps are one of the most productive; their flexibility means they can be used on mobile devices such as smartphones and tablets, and they can help develop mobile, ubiquitous learning inside and outside school. Gamified app design for education is a field of continuous experimentation conditioned by variables of student age, educational stage, and the content and competences to be developed. The gamified app design for higher education is not the same as for business or for students in primary education. App design for education is determined by a set of didactic, curricular, motivational, and technical principles. Most published scientific evidence shows that if gamified educational processes are solidly designed, they boost students’ motivation, commitment, and academic outcomes (Zainuddin et al., 2020).

There is no doubt that the choice of a good educational app depends on different variables that teachers value when making their choice (Papadakis, 2021; Taylor et al., 2022). One of the variables that affect the choice of an app is its presentation in Apple or Google app stores, which is influenced by the visual information provided by the app more than by the written information (Dubé et al., 2020). In this sense, it is important to note that the educational apps category does not always include apps with high educational value (Papadakis, 2021; Taylor et al., 2022). In addition, there are different frameworks for evaluating educational apps (Kolak et al., 2021; Meyer et al., 2021) and among the technical variables that have been proposed to evaluate the quality of educational apps, some important variables have been proposed: manipulability (Highfield & Goodwin, 2013) and usability (Walker, 2011). One of the best-known frameworks for app evaluation is from Hirsh-Pasek et al., (2015a, b) which includes four dimensions: active, engaged, meaningful, and social. More recently Dubé et al. (2020) proposed five dimensions to evaluate the quality of an app from the point of view of what students should learn: curriculum-based content, scaffolding and immediate feedback, pedagogical framework used and developed by or with educational experts. Despite these references, the strategy usually used by teachers when choosing an app is the external evaluation of web pages that assess the quality of apps (Educación3.^0^., 2023) and in this evaluation, special attention is paid to the following dimensions (Montazami et al., 2022, p. 3): age appropriateness, easy of play, inappropriate content (violence, sex, language, drinking, drugs, smoking), monetization (cost and advertisements), privacy, and user reviews.

Furthermore, to achieve this, gamified app design must adhere to a set of principles that combine the gaming and educational dimension by linking the experience of the game to learning, without infringing users’ rights on personal data protection privacy, and promoting safe and ethical use of the devices (Krath et al., 2021). Nevertheless, there is still no general consensus on the supposition that gamified apps have a positive influence on academic performance, as various meta-analyses have shown (Wouters et al., 2013). In the end, a teacher will adopt, or reject, a gamified app as an educational resource according to a range of variables and conditioning factors that enhance or inhibit their use in the classroom, and which in turn condition their design.

The scientific literature has been identifying in recent years different variables that affect the adoption of gamification in the classroom (Adams & Clark, 2014; Araujo & Carvalho, 2017; Lee & Hammer, 2011). Although these studies focus on the incidence of gamification regardless of devices, resources or applications. In this sense, after the COVID-19 Pandemic new free digital resources have been proposed to support the continuation of teaching and learning, some of them as educational apps have been identified as powerful resources to complement textbooks and learning, such as “Brac Education Progamme” or the “Kukua Package” of game-based apps to enable children to teach themselves how to read, write and do basic numeracy (OECD, 2020). In this study, we have proposed three dimensions (Table 1) that have been identified to foster the educational effectiveness of gamified apps: (1) cognitive scaffold: operational experience and feedback; (2) curriculum connection and (3) digital sustainability and accessibility.

**Table 1:** Selected dimensions and attributes for app design measurement

Dimensions	Variables	Items	Authors
Curriculum Connection	Curriculum Connection	CC1. An educational gamified app should comtemplate the development of specific competencies CC2. An educational gamified app should comtemplate the development of transversal competencies CC3. An educational gamified app should comtemplate the development of curricular contents of one or several subjects CC4. An educational gamified app should comtemplate the development of education in values CC5. An educational gamified app should integrate learning situations	Hirsh-Pasek et al., (2015a, b); Lynch and Redpath (2012); Falloon (2013)
Cognitive Scaffold	Feedback and operational experience on children’s game experiece and learning	FO1. An educational gamified app should reinforce cognitive functions (informative, completion, corrective, differentiation, restructuring) FO2. An educational gamified app should reinforce metacognitive functions (informative, specification, corrective, guiding) FO3. An educational gamified app should be motivational (incentive, task facilitation, self-efficacy enhancing, and reattribution) FO4. An educational gamified app should promote self-regulated learning and adaptive scaffolding FO5. An educational gamified app should be based both on individual and collaborative processes FO6. The educational gamified app has to provide personalised an adaptive feedback to improve students’ performance and to support student reflection	Shute & Ventura (2013); Deterding et al., (2011). Hamari (2017); Buckley and Doyle (2016); Hamari (2017)
Cognitive Scaffold	Assessment and Learning Analytics	AS1. The overcoming of challenges in the app should be related to the overcoming of the curriculum evaluation criteria AS2. The educational gamified app has to provide information about students’ performance to parents and teachers AS3. The educational gamified app has to provide tips to partents to help and control students activity in the app AS4. The educational gamified app has to provide scales of progress	Lockyer et al. (2013); Admiraal et al. (2020); Almohammadi et al. (2017); Bernhardt (2017); Charitopoulos et al. (2020); Ifenthaler, D. (2021);
Digital sustainability	Sustainable and protection of personal data	PD1. App informs on the protection of personal data PD2. App explains why it asks for permission PD3. App applies an efficient cache policy PD4. App optimizes the use of location services PD5. App makes decision reversal easyly PD6. App promotes a safe, flexible and collaborative learning environment PD7. App guarantees transparency (algorithms and processes)	Baker and Hjarlmarson (2019); Regulation (EU) 2016/679; Feiler et al. (2018); Ooijen and Vrabec (2019)
Accessibility	Equal access	EA1. App design ensures that everybody has access to all functionalities EA2. App optimizes media and images EA3. App implements Zoom/Magnification (resize text) EA4. App is designed in a consistent layout EA5. App allows keyboard control for touchscreen devices	Patch et al. (2015); Directive 2016/2102
Operational experience and feedback	Flow: Design and balance	FD1. App should be designed with clear goals FD2. App should be designed with the implementation of badges, quests, points and levels FD3. App should be designed with a balance of skill and challenge FD4. App should be designed with immersive and intrinsically motivated experiences	Csikszentmihalyi (2014); Özhan and Kocadere (2020); Wang et al. (2016)
Efficacy	Learning efficacy	EF1. I believe that the use of an educational gamified app can help me to teach with greater efficacy EF2. I believe that an educational gamified app can help students to acquire more knowledge EF3. I believe that an educational gamified app increases students’ motivation to learn	Chau (2014); Falloon (2013); Chiong (2012)
Satisfaction	Satisfaction with an gamified app	S1. I am satisfied with the use of an educational gamified app to support the teaching process S2. I am satisfied with the mobile and ubiquitous learning S3. An educational gamified app satisfies students’ learning needs	Lee (2010); Lee et al. (2009)

### Cognitive scaffold: operational experience and feedback

The potential for cognitive development is one of the main variables that influence take-up of a gamified app in the classroom, and as a complementary tool for school work outside the classroom. Teachers who use gamified apps emphasize gaming’s potential for interaction by trial and error processes, and for stimulating interest in curricular content (Demirbilek & Tamer, 2010; Sánchez-Mena & Martí-Parreño, 2017). Teachers also appreciate such apps for sharpening understanding of concepts by additional visual input, for developing creativity and capacity for processing content (Demirbilek & Tamer, 2010), and for their potential for developing life skills such as sociability, cooperation, collaboration and team work (Smith, 2018). Gamified resources are also appreciated for promoting alternatives to traditional learning, by varying the presentation of content and placing it within more inclusive learning environment (Jedel & Palmquist, 2021). In this sense, authors such as Sun et al. (2018) consider that learning assistance tools used with digital games can help students to create problem-solving strategies while supporting achievement.

These gamified mobile learning approaches relate different elements, such as fantasy, stories, curiosity, feedback, competition, modality, and interaction, that are promising features to enable learning (Araujo & Carvalho, 2017). This conjuction of factors not only enables cognitive development in content in a particular school subject, but also contributes significantly to general cognitive development and complements it with an affective element that can also have a beneficial effect on student behavior (Clark et al., 2016). Gamified digital environments give students access to a wide variety of materials and multimodal information that they can use to surmount challenges and solve problems, all within the experience of the game; this dynamic encourages the student to learn and direct their learning strategies towards managing situations by applying competences, learning structures and skills needed in problem solving (Wouters et al., 2013). Cognitive scale formats can also be complemented by different game designs and formats; some studies (Wouters et al., 2013) have shown that formats that encourage students to request external support for solving problems or challenges bolster their cognitive development with the active use of feedback, guiding, prompts or structures. And when the design of the gamified experience allows the student, teacher and family to access the learning analytics, student commitment and involvement is enhanced in terms of the game and the challenges it presents, linked to subject content, leading to stronger cognitive development (Admiraal et al., 2020; Almohammadi et al., 2017; Bernhardt, 2017; Charitopoulos et al., 2020).

Other studies show that when an intelligent digital tutor is incorporated into the game experience, a presence that can be consulted in the problem-solving process and for doubts that arise during the game linked to curricular content, it fosters the student’s procedural and cognitive development. Pareto et al. (2012) found that students who could access this type of support in a 2-D Mathematics game saw their Mathematics test scores rise. A study by Vázquez-Cano et al. (2021) showed positive results when a gamified chatbot support was on hand for students learning about Spanish Language punctuation, with university access exam scores for Spanish Language improving substantially. Adams and Clark (2014) presented a study in which a virtual agent that provided complementary explanations for students in a gamified experience in Physics got higher scores in that subject. O’Neil et al. (2014), analyzing students participating in a Mathematics puzzle game, showed that external orientation processes facilitated the connection between in-game summaries and concepts, which had a strong positive impact on these students’ test scores in the subject. This type of adaptive feedback via a system of targeted stimuli within the gaming experience is shown to be effective in boosting students’ academic performance (Chen et al., 2019).

One of the key aspects in designing a gamified environment app with this type of adaptive feedback is that the game experience should flow seamlessly, unimpeded or slowed to the point where the student finds the game dynamic unattractive and switches off (Adams & Clark, 2014; O’Neil et al., 2014). This adaptive feedback should not be intrusive but encourage the student to use it when stuck or unable to advance without seeking assistance (Shute & Ventura, 2013). This in-game assistance must also feature in the reports generated for teachers and families so that a learning narrative can be created to allow for external methods of reinforcement, broadening and deepening the knowledge to be developed (Vázquez-Cano & Sevillano, 2021), and to generate a design that is adapted to the challenges and sequences of the game within the app (Ronimus et al., 2014). Adaptive feedback is useful as it adds flexibility to the gaming experience and associates it to academic achievement in particular content and competences, by enriching the gaming experience (e.g.: question prompts, material presentation, feedback) (Law & Chen, 2016). Nevertheless, some recent studies, such as the meta-analysis by Liu et al., (2021a, b), showed that adaptive scaffold can spoil the student’s experience of the game and hinder their engagement. To implement a true “Learning engagement” three areas related to “engagement” must be properly combined: cognitive (mental effort and cognitive strategies) (Greene, 2015), emotional (enjoyment and excitement) (Sailer et al., 2017), and behavioural (students’ participation) (Goggins & Xing, 2016).

When the game experience is well designed, researchers find that the influence of games on the educational processes is positive, with statistically significant improvements demonstrated in motivation and commitment (Squire & Jenkins, 2003). In this sense, the following research in primary education (Uluyol & Sahin, 2016) confirms an improvement in motivation when gamification forms part of the teaching–learning process.

### Curriculum connection

One of the fundamental variables that conditions the successful application of a gamified app to student’s work inside and outside the classroom is the strength of its connection to the curriculum in terms of content, competences, values and assessment. When teacher and family perceive that the game is ethical, safe and responsible, and that the experience is clearly linked to the curriculum, then the chance of its adoption as a successful addition to traditional school work increases (Vázquez-Cano et al., 2022). A condition for successful curricular connection relates to the particular subject or content. The effectiveness of a gamified process in ubiquitous contexts will depend largely on the type of subject and content that has been gamified (Demirbilek & Tamer, 2010; Sánchez-Mena & Martí-Parreño, 2017). Another condition is timing, in other words, the moment in the development of the curriculum when the app is introduced. Several studies have shown that gamification processes are most effective when used sparingly, not continuously, for example during assessment phases or for homework. It has also been shown that introducing a gamified app following presentation of content and strategies in class is more effective (Mee Mee et al., 2020; Pektas & Kepceoglu, 2019; Smith, 2018).

When the type of content is suitable and the time to right to introduce the app, a third variable that influences successful application of a gamified app will be that there is a clear connection to the objectives and assessment criteria for the educational stage and age of the students, and that their level of competence, attitude, knowledge and skills in the subject areas being developed can be easily verified (Fombona et al., 2020; Kingsley & Grabner-Hagen, 2015; Parra-González et al., 2020; Vázquez-Ramos, 2021). The connection between game and curriculum can be made from various perspectives, as long as they enable the student’s progress to be continuously monitored, for example, when the game includes roadmaps to reach objectives, different levels of complexity, with text and visual resources to enable assimilation of concepts and reinforcement of key elements to build an exhaustive knowledge of the material (Jedel & Palmquist, 2021).

Another potentially inhibitating variable is the lack of knowledge of the didactic functionality and suitability of these apps for the curriculum. Several authors have shown that when there has been rigorous assessment of such resources, the likelihood of adoption by teachers increases, for example, when gaming apps are successfully matched to the pedagogical needs of particular context (Green et al., 2014; Papadakis et al., 2017), or when analytical models or frames of reference are used to enable the design and adaptation of gamified didactic proposals (Tenório et al., 2020; Vázquez-Ramos, 2021). Green et al. (2014) developed an instrument to check digital applications’ pedagogical suitability as support for the curriculum. The model’s pedagogical bases examine the app’s authenticity and procedures, the possiblity of personalizing the app (self-paced, self-regulation, customisation) and potential for student collaboration, as a key component of learning. Other recent models that evaluate gamified resources and their suitability for curricular development include one by Papadakis et al. (2017) which focuses on measuring the practical effectiveness of apps in relation to educational content, thereby enabling teachers to decide on the suitability of an app, given the lack of pedogogical justification of many of the apps on the market.

### Digital sustainability and accessibility

One of the fundamental aspects that conditions the use and design of educational apps is their security and ethics in data protection issues. This aspect is very sensitive in all educational stages, but especially in primary education. The sustainability of the use of devices is an essential aspect for its implementation in teaching. In this sense, different researches have been carried out in this regard, for example, Reyes et al. (2018) analyzed 5,855 free children's games from the point of view of data security for the user, concluding that most of them violated the principles established by COPPA (Children's Online Privacy Protection Act), such as tracking and behavioral advertising and noting that “the 19% of children's apps collect identifiers or other personally identifiable information via “Software Development Kits” whose terms of service outright prohibit their use in child-directed apps” (Reyes et al., 2018). One of the areas in which data protection is most complicated and decisive is in the treatment of learning analytics, which already represent a threat to autonomy and privacy (Parsons, 2021). This is essential to guarantee the privacy of students regarding such a sensitive information as their academic performance (Willis et al, 2016). Likewise, there do not seem to be many differences between supposedly free and paid apps in terms of data access, collection and transmission (Han et al, 2019).

Another aspect that significantly conditions the effectiveness of a gamified app is its accessibility. It is important and crucial that digital devices and apps can be used by any type of student regardless of their functional diversity. In this sense and according to the recommendations of the World Wide Web Consortium (W3C) it is necessary that they comply with the Web Content Accessibility Guidelines (WCAG). In Patch et al. (2015) describe the principles that should guide the design and usability of applications and resources with mobile accessibility, these are: 1) Perceivable (small screen size, zoom/magnification and contrast), 2) Operable (keyboard control for touchscreen devices, touch target size and spacing, touchscreen gestures, device manipulation gestures and placing buttons where they are easy to access), 3) understable (changing screen orientation (portrait/landscape), consistent layout, provide instructions for custom touchscreen and device manipulation gestures, among others), (4) Robust (set the virtual keyboard to the type of data entry required, provide easy methods for data entry and support the characteristic properties of the platform).

Based on these theoretical principles, this study establishes the following research question: “What design elements of gamified apps favor their educational effectiveness and their use by primary school teachers?”.

## Method

There are various proposals that set out to assess and quantify the perceived quality of a product or service once used by a consumer. Some are based on constructs of the expectations generated by the user (Martínez-Tur et al., 2005) and others focus on the importance attributed to each element of those constructs (Martilla & James, 1977), the latter known as Importance-Performance Analysis (IPA). IPA aims to identify the value alloted by users in terms of the importance and performance of a series of quality criteria. IPA is based on theoretical proposals from multi-attribute and value-expectation models (Abalo-Piñeiro et al., 2006). As Abalo-Piñeiro et al. (2006, p. 730) stated: “These models sustain that each service is composed of a series of independent attributes, and that consumer attitudes are formed by the weighted aggregate of the assessments of each of these attributes, such that it is necessary to analyze in detail all the elements that configure a service.”

**Fig. 1:**
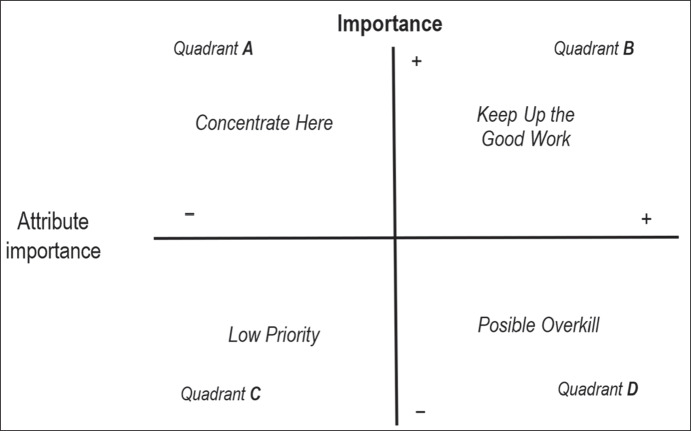
Importance Performance Analysis Matrix. Source: adapted from Martilla and James (1977)

This model has been applied to a range of areas and environments including education (Huybers, 2014; Siniscalchi et al., 2008). One of the main attractions of IPA is that the results can be displayed in a two-dimensional graph form that yields four quadrants (Fig. 1): (a) “concentrate here” (high importance, low valuation), which highlights the most important aspects of the organization for the client, but where performance is deemed insufficient; (b) “keep up the good work” (high importance, high valuation); (c) “low priority” (low importance, low valuation); (d) “possible overkill” (low importance, high valuation), presenting aspects of the organization that are performed well but which are unimportant for the users.

The approach adopted in this research was formulated from the adaption of the following expression (Fishbein & Ajzen, 1975):

$$Vos\sum_{i=1}^{n}{{I}_{i} V}_{i}$$

where

*Vos* is the global evaluation of the app in terms of digital sustainability;

*I* is the importance attached to each attribute of digital sustainability by the teachers:

*V* is the evaluation that each attribute receives;*n* is the number of attributes that constitutes the digital sustainability setting.

Based on this model, and considering that users normally judge performance according to a limited number of characteristics (Edwards & Newman, 1983), such that the most important attributes will largely affect users’ evaluation while the least important will scarcely influence the global evaluation (Swan & Combs, 1976), we were able to obtain an indirect measure of the elements of digital sustainability that encourage or discourage the adoption of educational apps to complement the development of the teaching–learning processes by teachers in primary education. We used the direct means as the best quantifiers of importance, as opposed to the indirect means such as those obtained by regression coefficients.

### Participants

The study sample consisted of 212 primary Spanish teachers from the 16 autonomous communities of Spain: 32.1% men and 67.9% women. The mean age of participants was about 34 (mean = 33.14, standard deviation = 2.41). These 212 teachers have used at least one educational app inside or outside the classroom to support their students' learning, so they have experience in the educational use of apps.

### Instrument and variables

The data were gathered between September 1st and October 21st (2021). A questionnaire was designed to be completed online by the teachers, having given prior informed consent, under the Spanish Research Project (PDC2022-133185-I00). The sample was heterogeneous, with participants teaching in different courses (n = 20-1^st^ / n = 22-2^nd^ / n = 20-3^rd^ / n = 50-4^th^ / n = 50- 5^th^ / n = 50- 6^th^) corresponding to students from 6 to 12 years old, and from different subjects: Spanish Language (32%), English Language (19%), Maths (28%), Social Sciences (8%) Natural Sciences (7%) and Physical Education (6%), in order to boost the study’s external statistical validity. The questionnaire was sent to the official email account of the different schools and consisted of eight latent variables with 37 items. The first six variables are related to the three dimensions of the theoretical framework. The teachers had to respond to each item by scoring it on a 1–7 scale, 1 meaning “totally disagree” and 7 “totally agree”. The items selected had been adapted from works by a range of authors (Table 1) and from previous research activities using focus groups with a a selection of teachers using gamified apps.

**Fig. 2:**
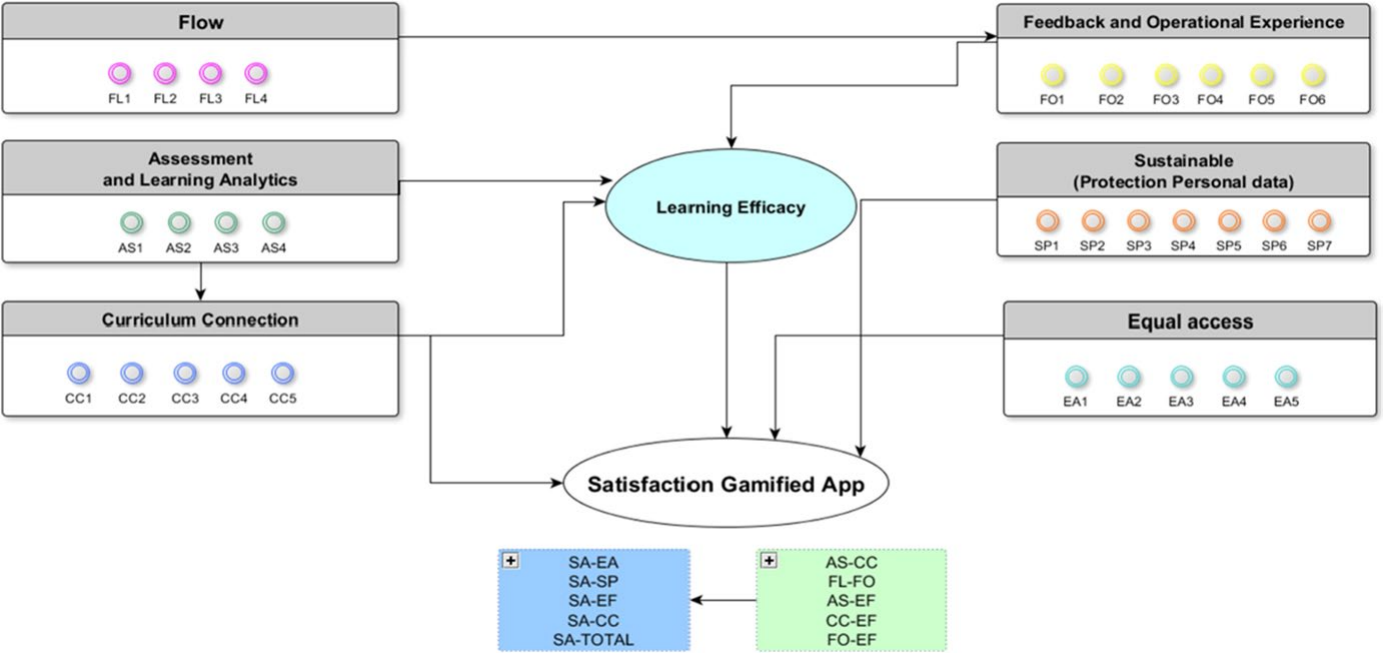
Model of structural equations specified for the derivation of the importance of atributes

In addition to these attributes, the data on importance were obtained by applying a structural equations model, for which the questionnaire included another latent variable, “satisfaction with app design”, defined on a 0–7 scale, in which 1 denoted “of lowest value” and 7 “of highest value”. The structural equations model is based on the principle that the characteristics related to app design (tecno-didactics issues distributed in six latent variables, see Table 1) have a direct effect on the satisfaction of teachers when using or recommending educational apps in the teaching–learning processes. This equation model was calculated with the AMOS software (Fig. 2).

## Results

Prior to structural equation modeling, an analysis of the data was carried out to test the validity and reliability of the scales. The Cronbach Alpha coefficient was above 0.8 and the Kaiser-Meyer index exceeded 0.78. The null hypothesis was rejected by the Bartlett sphericity test, thus enabling factor analysis. Factor analysis yielded seven factors with the varimax rotation method, thus confirming the validity of the questionnaire. The maximum likelihood estimation method was used to calculate the structural model’s parameters. Although the data did not conform to the multivariate normal distribution, this method facilitated the convergence of estimates (Lévy et al., 2006). The model was assessed on criteria from Bollen (1989) and Rindskopf and Rose (1988), who proposed that the measurement model and structural model be evaluated separately. The measurement model’s validity and reliability were analyzed, the latter in terms of the reliability of the items and of each construct. For validity, both convergent and discriminant validity were analyzed, with the results shown in Tables 2 and 3.

**Table 2:** Standardized estimations

Factors			λ	Cronbach’s α	CR (Composite Reability)	AVE
*Curriculum Connection*				0.923	0.901	0.755
CC1		Curriculum Con	0.843			
CC2		Curriculum Con	0.834			
CC3		Curriculum Con	0.811			
CC4		Curriculum Con	0.789			
CC5		Curriculum Con	0.823			
*Feedback and Operational Experience*				0.911	0.902	0.814
FO1		Feedback and op. experience	0.787			
FO2		Feedback and op. experience	0.866			
FO3		Feedback and op. experience	0.815			
FO4		Feedback and op. experience	0.889			
FO5		Feedback and op. experience	0.811			
FO6		Feedback and op. experience	0.865			
*Assessment and Learning analytics*				0.944	0.851	0.712
AL1		Assessment and L. Analytics	0.821			
AL2		Assessment and L. Analytics	0.832			
AL3		Assessment and L. Analytics	0.822			
AL4		Assessment and L. Analytics	0.818			
*Sustainable (Protection Personal data)*				0.923	0.944	0.881
SP1		Sustainable (Prot. Personal Data)	0.934			
SP2		Sustainable (Prot. Personal Data)	0.889			
SP3		Sustainable (Prot. Personal Data)	0.921			
SP4		Sustainable (Prot. Personal Data)	0.896			
SP5		Sustainable (Prot. Personal Data)	0.878			
SP6		Sustainable (Prot. Personal Data)	0.923			
SP7		Sustainable (Prot. Personal Data)	0.899			
*Equal access*				0.833	0.901	0.811
EA1		Equal access	0.887			
EA2		Equal access	0.900			
EA3		Equal access	0.812			
EA4		Equal access	0.834			
EA5		Equal access	0.889			
*Flow*				0.932	0.901	0.889
FL1		Flow	0.911			
FL2		Flow	0.905			
FL3		Flow	0.923			
FL4		Flow	0.907			
*Efficacy*				0.931	0.919	0.798
EF1		Efficacy	0.901			
EF2		Efficacy	0.897			
EF3		Efficacy	0.838			
*Satisfaction with of an gamified app*				0.948	0.938	0.833
SA1		Satisfaction	0.901			
SA2		Satisfaction	0.923			
SA3		Satisfaction	0.971

**Table 3:** Discriminant validity of measures

	CC	FO	AL	SP	EA	FL	EF	SA
CC	**0.878**							
FO	0.534	**0.814**						
AL	0.551	0.605	**0.805**					
SP	0.612	0.599	0.699	**0.855**				
EA	0.589	0.556	0.678	0.586	**0.871**			
FL	0.711	0.587	0.589	0.702	0.555	**0.787**		
EF	0.721	0.591	0.556	0.713	0.578	0.761	**0.791**	
SA	0.741	0.731	0.665	0.595	0.798	0.613	0.600	**0.901**

*The bold numbers of the diagonal are the square root of the AVE. Off-diagonal elements are correlations between constructs. CC: Curriculum Connection. FO: Feedback and operational experience. AL: Assessment and Learning Analytics. SP: Sustainable and protection of personal data. EA: Equal access. FL: Flow. EF: Learning efficacy. SA: Satisfaction*

Table 2 shows that the reliability of the items is verified. In terms of the reliability of the constructs, all the values for the Cronbach α coefficient and the Composite Reliability (CR) coefficient exceed 0.7, which confirms the reliability of the constructs. Table 2 also shows that the average variance extracted (AVE) is above 0.5, which verifies the constructs’ convergent validity. Table 3 presents the results for discriminant validity. For this, the correlation matrix between the constructs was calculated, which confirmed that the correlations were less than the AVE square root.

We observed that all correlations between the constructs amount to less than the corresponding AVE values for each construct, thus confirming that the factors measure different concepts. Finally, in assessing the structural model, it was found that the estimated value of the squared coefficient of multiple correlation for each dependent or endogenous construct exceeds 0.5, and that the factor loads between constructs is significant. Table 4 summarizes the structural model and the hypothesis testing results.

**Table 4:** Parameter estimates

Relation			Estimate	S.E	C.R	Standardized estimate	*p*
AS		CC	0.567	0.051	15.896	0.565	***
FL		FO	0.501	0.053	15.321	0.810	***
AS		EF	0.709	0.101	11.781	0.601	***
CC		EF	0.401	0.060	12.431	0.541	***
FO		EF	0.517	0.041	9.451	0.689	***
SA		EA	0.586	0.053	7.621	0.610	***
SA		SP	0.612	0.061	9.301	0.502	***
SA		EF	0.357	0.100	14.341	0.789	***
SA		CC	0.610	0.045	15.001	0.581	***
CC1		CC	1			0.843	
CC2		CC	0.833	0.102	25.567	0.923	***
CC3		CC	0.868	0.031	21.456	0.899	***
CC4		CC	1.451	0.071	19.001	0.812	***
CC5		CC	1.271	0.063	20.178	0.871	***
FO1		FO	1			0.787	
FO2		FO	1.201	0.600	16.189	0.901	***
FO3		FO	1.205	0.055	15.003	0.837	***
FO4		FO	0.913	0.201	16.111	0.834	***
FO5		FO	1.401	0.071	15.562	0.886	***
FO6		FO	1.108	0.057	9.649	0.891	***
AL1		AL	1			0.821	
AL2		AL	0.911	0.117	17.300	0.889	***
AL3		AL	1.357	0.043	9.643	0.917	***
AL4		AL	0.919	0.057	12.134	0.873	***
SP1		SP	1			0.934	
SP2		SP	1.111	0.055	13.893	0.912	***
SP3		SP	1.054	0.132	15.491	0.877	***
SP4		SP	1.301	0.063	8.203	0.854	***
SP5		SP	1.110	0.071	16.397	0.891	***
SP6		SP	0.911	0.051	16.001	0.910	***
SP7		SP	1.012	0.055	15.107	0.831	***
EA1		EA	1			0.887	
EA2		EA	1.006	0.121	11.101	0.876	***
EA3		EA	1.131	0.072	10.845	0.818	***
EA4		EA	1.145	0.063	14.145	0.895	***
EA5		EA	0.998	0.101	8.895	0.913	***
FL1		FL	1			0.911	
FL2		FL	1.018	0.087	16.117	0.799	***
FL3		FL	1.045	0.099	10.009	0.810	***
FL4		FL	1.023	0.123	14.176	0.805	***
EF1		EF	1			0.901	
EF2		EF	1.112	0.079	16.111	0.789	***
EF3		EF	1.002	0.011	11.856	0.806	***
SA1		SA	1			0.901	
SA2		SA	1.001	0.013	32.145	0.819	***
SA3		SA	1.021	0.018	35.349	0.845	***

Finally, Table 5 presents the values of the structural model’s fitness indices. All the measures fall within the established limits, which confirm the data’s goodness of fit.

**Table 5:** Fit indices for the structural equations model

Fit index	Actual
χ^2^ df Goodness-of-fit index (GFI) Adjusted goodness-of-fit-index (AGFI) Comparative fit index (CFI) Root mean square error of approximation (RMSEA) Normed fit index (NFI) Non-normed fit index (NNFI) Parsimony normed fit index (PNFI)	321.101* 1.756 .801 .812 .053 .060 .900 .910 .783

We calculate the importance of each attribute according to the criteria established by Allen et al. (2020), the importance derives from the total of the effects of each latent variable on the satisfaction with the app design (Table 6).

**Table 6:** Predictor variables on satisfaction

	Direct effect	Indirect effect	Total effect
CC	0.000	0.770	0.770
FO	0.000	0.677	0.677
AL	0.699	0.000	0.699
SP	0.267	0.246	0.513
EA	0.000	0.601	0.601
FL	0.358	0.423	0.781
EF	0.301	0.342	0.643

The relative importance of each attribute was calculated by multiplying the total effects of each latent variable by the standardized regression weight (Table 7).

**Table 7:** Calculation of importance

Atribute	Total effect	Standarized Coefficients	Importance
CC1	0.770	0.843	0.64
CC2		0.834	0.61
CC3		0.811	0.65
CC4		0.789	0.50
CC5		0.823	0.58
FO1	0.677	0.787	0.59
FO2		0.866	0.57
FO3		0.815	0.55
FO4		0.889	0.65
FO5		0.811	0.63
FO6		0.865	0.69
AL1	0.699	0.821	0.68
AL2		0.832	0.60
AL3		0.822	0.52
AL4		0.818	0.58
SP1	0.513	0.934	0.64
SP2		0.889	0.61
SP3		0.921	0.42
SP4		0.896	0.40
SP5		0.878	0.57
SP6		0.923	0.65
SP7		0.899	0.56
EA1	0.601	0.887	0.45
EA2		0.900	0.51
EA3		0.812	0.43
EA4		0.834	0.48
EA5		0.889	0.35
FL1	0.781	0.911	0.40
FL2		0.905	0.62
FL3		0.923	0.51
FL4		0.907	0.64
FL1		0.911	0.64
FL2	0.645	0.905	0.61
FL3		0.923	0.65
EF1		0.901	0.50
EF2	0.643	0.897	0.58
EF3		0.838	0.59

The values obtained show that importance ranges between 0.29 (lowest) and 0.67 (highest), while the range of satisfaction is between 2.71 (lowest) and 4.13 (highest). According to Ormanović et. al. (2017, p. 60), discrepancy is calculated as the difference between normalized performance and importance (still known as the gap between the expectations and perceptions of service/products by users). Since IPA requires data on the same scale, a normalization between 0.00 and 1.00 has been performed to obtain the normalized values. The 31 items in Table 8 correspond to the first 31 items of Table 1 (First six latent variables).

**Table 8:** Normalized Importance, satisfaction and discrepancies

Items	Norm. Import	Norm. Satis	Discrep
1. An educational gamified app should comtemplate the development of specific competencies	0,80	0,91	-0.11
2. An educational gamified app should comtemplate the development of transversal competencies	0,98	0,97	0.01
3. An educational gamified app should comtemplate the development of curricular contents of one or several subjects	0,66	0,61	0.06
4. An educational gamified app should comtemplate the development of education in values	0,60	0,34	0.26
5. An educational gamified app should integrate learning situations	0,63	0,77	-0.14
6. An educational gamified app should reinforce cognitive functions (informative, completion, corrective, differentiation, restructuring)	0,70	0,81	-0.09
7. An educational gamified app should reinforce metacognitive functions (informative, specification, corrective, guiding)	0,65	0,38	0.27
8. An educational gamified app should be motivational (incentive, task facilitation, self-efficacy enhancing, and reattribution)	0,35	0,49	-0.14
9. An educational gamified app should promote self-regulated learning and adaptive scaffolding	0,54	0,89	-0.35
10. An educational gamified app should be based both on individual and collaborative processes	0,46	0,54	-0.08
11. The educational gamified app has to provide personalised an adaptative feedback to improve students’ performance and to support student reflection	0,78	0,54	0.24
12. The overcoming of challenges in the app should be related to the overcoming of the curriculum evaluation criteria	0,45	0,67	-0.22
13. The educational gamified app has to provide information about students’ performance to parents and teachers	0,48	0,61	-0.13
14. The educational gamified app has to provide tips to partents to help and control students activity in the app	0,59	0,67	-0.08
15. The educational gamified app has to provide scales of progress	0,34	0,67	-0.33
16. App informs on the protection of personal data	0,56	0,91	-0.35
17. App explains why it asks for permission	0,90	0,67	0.23
18. App applies an efficient cache policy	0,70	0,59	0.11
19. App optimizes the use of location services	0,67	0,17	0.50
20. App makes decision reversal easyly	0,61	0,41	0.20
21. App promotes a safe, flexible and collaborative learning environment	0,54	0,67	-0.13
22. App guarantees transparency (algorithms and processes)	0,31	0,89	-0.58
23. App design ensures that everybody has access to all functionalities	0,41	0,65	-0.24
24. App optimizes media and images	0,36	0,23	0.13
25. App implements Zoom/Magnification (resize text)	0,23	0,17	0.06
26. App is designed in a consistent layout	0,12	0,59	-0.47
27. App allows keyboard control for touchscreen devices	0,39	0,31	0.08
28. App should be designed with clear goals	0,71	0,67	0.04
29. App should be designed with the implementation of badges, quests, points and levels	0,67	0,76	-0.09
30. App should be designed with a balance of skill and challenge	0,67	0,56	0.11
31. App should be designed with immersive and intrinsically motivated experiences	0,80	0,91	-0.11

Norm. Import. (Normalized Importance) / Norm. Satis. (Normalized Satisfaction) / Discrep. (Discrepancies).

**Fig. 3:**
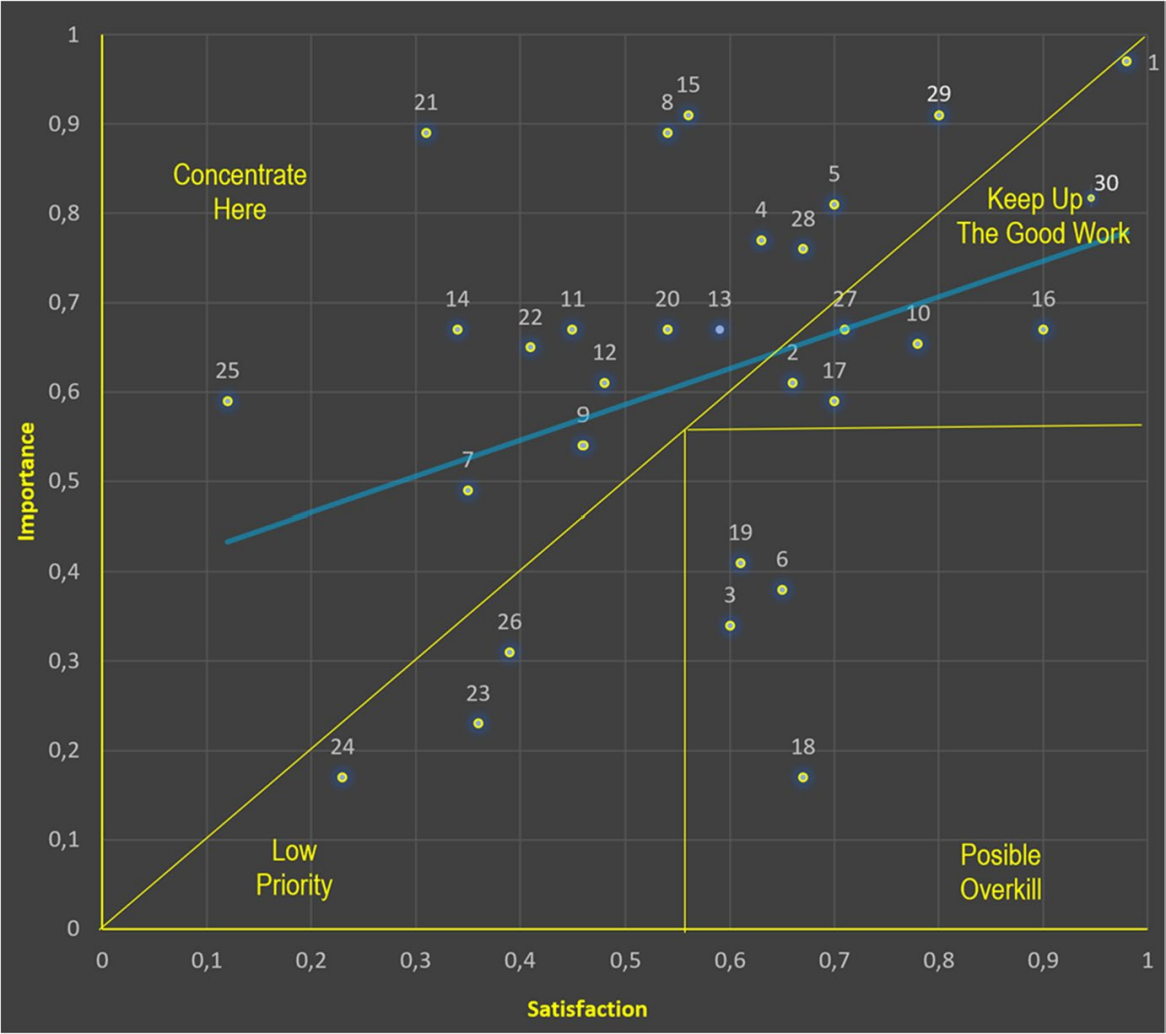
Representation of combined classic and diagonal models

With the values obtained in Table 8, the IPA representation was performed (Fig. 3). The attributes with positive discrepancies are those in which importance exceeds satisfaction, thus, they represent a high priority for improvement.

In Fig. 3, it can be seen in quadrant A “concentrate here”, the main elements for the design and adoption of a gamified app are: (5) integration of learning situations, (11) provide personalised an adaptative feedback, (9) promotion of self-regulated learning and adaptive scaffolding, (13) provide information about students’ performance to parents and teachers, (12) related to the overcoming of the curriculum evaluation criteria, (15) explain why app asks for permission, (22) guarantee transparency (algorithms and processes), 30) designed with immersive and intrinsically motivated experiences, (28) implementation of badges, quests, points and levels.

In this line, teachers consider that some issues need to keep up the good work (Quadrant B): (2) development of transversal competencies, (10) based both on individual and collaborative processes, (16) inform on the protection of personal data (17) explain why app asks for permission and (27) designed with clear goals and (30) balance of skill and challenge. Furthermore, we observe that there are three attributes considered of “low priority” (Quadrant C): (23) access to all functionalities. (24) optimize media and images (26) keyboard control for touchscreen devices. Four elements are considered as a “possible overkill” (Quadrant D): (3) development of curricular contents of one or several subjects. (6) reinforce cognitive functions (informative, completion, corrective, differentiation, restructuring), (18) efficient cache policy and (19) use of location services.

## Discussion

The results showed that the teachers consider a good gamified apps across six dimensions, in which the app should: (1) establish a clear link between the game and curricular content and competence development; (2) promote self-regulated learning through individual and collaborative activities; (3) offer adapted learning by integrating differentiated personalized learning pathways; (4) integrate learning analytics that can be consulted by teacher, student and family; (5) comply with data protection regulation and promote a safe, sustainable and ethical use of the information generated; (6) take into account different levels of functional diversity. When the gamified app design incorporates these attributes, primary education teachers perceive that such resources can be integrated effectively into the teaching–learning processes.

These results complement other traditional studies in which only three priority areas were established in the design of activities based on digital games: cognitive, emotional, and social (Lee & Hammer, 2011). One of the areas considered necessary for the adoption of a gamified app in primary education is represented by Dimension 1: “Curriculum Connection”. For a gamified app to acquire high educational value, it must provide a strong curriculum connection, test key competencies, and enhance self-regulated learning (Adams & Clark, 2014; Baker & Hjarlmarson, 2019; Hirsh-Pasek et al., 2015a, b; Lynch & Redpath, 2012). The link between the design of the app and how it functions with the different parts of the curriculum (content, competencies, and assessment criteria) is a predictor of use and enhances adoption for use both inside and outside the classroom. Teachers are reluctant to use digital apps and devices when they perceive no clear connection to the content material they wish to develop in class (Falloon, 2013; Vázquez-Cano et al., 2022).

The results obtained have also confirmed that teachers consider that the cognitive area is important, and the activities associated with the game must be presented in non-linear sequences that allow the student to choose between different strategies which are the most appropriate depending on their progress and competence in the game. In this sense, Dimension 2: “Feedback and Operational Experience” is essential for the design of gamified apps. For this is necessary to promote self-regulated learning and adaptive scaffolding, through both individual and collaborative processes, and provide personalised adaptive feedback to improve students’ performance and to encourage student reflection. Different studies have been demonstrating the need to establish learning guidelines in which students have to use different rules that could be classified into levels to complete the balance scale task (Schrauf et al., 2011; Siegler, 2005). The app design must stimulate the development of individual and collaborative attitudes and student reflection, as well as offering a range of personalized learning pathways (Buckley & Doyle, 2016; Clark et al., 2016). These aspects are fundamental for an educational app and have been highlighted in several studies (Shute & Ventura, 2013).

Another area that needs close attention to ensure good educational app design is Dimension 3: “Assessment and Learning Analytics”: (a) the app should comply with curriculum evaluation criteria, and (b) must provide information on students’ performance to parents and teachers. The evaluation and learning analytics processes are fundamental in education today (Admiraal et al., 2020; Lockyer et al., 2013); there is an increasing need for apps that can automatically assess students’ work and provide guidance (Almohammadi et al., 2017; Bernhardt, 2017), to alleviate teacher workload, allowing them to concentrate on other didactic and methodological elements, and to provide educational support (Goggins & Xing, 2016). An app or computer program that can directly evaluate student’s work, in line with the curriculum’s evaluation criteria, enables teachers to provide guidance and feedback within the teacher-learning process, propose new more personalized learning pathways and keep families informed on how to guide their children’s learning process from home (Bernhardt, 2017; Charitopoulos et al., 2020). Automated evaluation processes and learning analytics of student performance are increasingly evident in numerous studies, as precursors and activators of student learning and facilitators of teachers’ work in the classroom (Ifenthaler, 2021). This type of assessment associated with the game should allow students to recover content and skills through the system of points or badges linked to the game and, in this way, to assist students with self-progress monitoring (Ackerman and Gross, 2020; Ryan et al., 2006).

These three categories have a direct link with Dimension 6: “Learning Efficacy”. Apps need to implement badges, quests, points, and levels into immersive and intrinsically motivated experiences. For digital devices to be effective in the classroom, they need to demonstrate that they can generate a lot of learning and are easy to use (Chau, 2014). Besides its curricular connection, a gamified app must also generate a gaming experience that motivates the user to scale the different levels of difficulty of curricular content and competences, so that the app is perceived by students and teachers as another viable element in the teaching–learning process (Chiong, 2012; Falloon, 2013).

Other fundamental aspects that can stimulate, or stifle, the development and application of devices and computer programs in education is digital sustainability and data protection, issues covered by Dimension 4. Today, compliance with national and international law makes it compulsory for digital devices to be safe, sustainable, and ethical to use in education or entertainment, especially in relation to devices used by minors (Feiler et al., 2018). The European framework covering educators’ digital competence, DigCompEdu sought to boost responsible use of technology in relation to devices, privacy and personal data protection, the physical and psychological well-being of the user, and protection of the environment (Regulation 2016/679); these areas also relate to the sustainable use of technology and sustainable development goals (Ooijen & Vrabec, 2019).

Dimension 5 refers to the requirement that app design ensures functional diversity, so that all students can use the app regardless of physical or cognitive impediment. This dimension focuses on promoting proper access to devices and computer programs; yet although the development of teaching–learning processes is crucial, not all teachers are aware of the different characteristics that such programs and apps must adopt in order to guarantee equal access to education for all, regardless of student typology (Patch et al., 2015). An educational app design must enable camera zooming, keyboard control and complete access to all functions in order to guarantee equal access to, and use of, digital devices and resources activated in the classroom (Directive 2016/2102).

It is important for teachers to get involved in app design to provide additional support to the work they develop in their particular subjects. Despite the abundance of apps in the market that link learning to play, compliance of app design criteria for use in formal education is by no means assured, as studies by Kingsley and Grabner-Hagen (2015), Fombona-Cadavieco et al. (2020) and Vázquez-Ramos (2021) have shown. It is essential to identify the transversal competences and content common to the entire curriculum that can be converted into digital formats, such as gamified apps. Transversal content such as calculus in Mathematics, spelling in Language, and the communication skills needed to practice first, and second languages can be converted into these formats. Although gamified apps can improve student learning, as they are portable, immediate, adaptable, and interactive, it is their pedagogical design that will ultimately determine their efficacy in use.

It must be considered that there are also a number of limitations associated with the use of gamified apps inside and outside the classroom. In this sense, it is necessary for the student to have devices connected to the network inside and outside the classroom, as well as the necessary competence for its educational use, as well as in security and data protection. Training programs should also be implemented so that teachers integrate devices and applications in an educational way for the development of skills and content associated with the curriculum. Likewise, on many occasions, the type of app and the cost of the licenses associated with its use will also have to be taken into account (Hill & Brunvand, 2018).

## Conclusion

The design and adoption of gamified apps in education is conditioned by a set of variables that can determine take-up or rejection of these resources for use inside and outside the classroom. Currently, there is a wide range of educational apps available, and their number seems to increase daily, yet teachers remain reluctant to adopt them as a resource to support and enhance the teaching–learning processes. In part, these misgivings are based on teachers’ perceived absence of an education-oriented design in the apps that allows them to be adapted to incorporate features of the subjects they teach, and to the content and competence requirements of such courses. Teachers consider that these apps can only be adopted in the teacher-learning process if they comply with solid pedagogical principles that include the facility to deliver feedback and self-regulated learning, adaptive systems that allow the student to opt for different learning pathways according to their level of achievement, and learning analytics for consultation by teacher, student, and family so that student progress and performance can be monitored. When a student plays on a gamified app, it is very important that the design provides activities that are sufficiently motivational, immersive, and challenging to stimulate the student to broaden and deepen their knowledge. These activities must connect to the assessment, content, and competence criteria in the curriculum. Teachers are increasingly aware of the importance that apps and digital devices comply with minimum standards of sustainability, and safe and ethical usage, and that data protection rights, in particular those of minors, are rigorously enforced. These aspects are fundamental to ensure that the learning taking place both inside and outside the classroom is supported by gamified educational apps that are effective, and that the psycho-emotional health and well-being of the students who use them are safeguarded.

## Limitations and recommendations for future study

The research is limited to a sample of Spanish teachers. It would be desirable that further studies in different socio-educational contexts could help to refute, clarify, or confirm these results. Likewise, the characteristics of teachers, students, families, and socio-educational contexts should be analyzed to define their influence on the adoption or not of this type of gamified apps in primary education.

## Data Availability

The datasets generated during and/or analysed during the current study are available from the corresponding author on reasonable request.
